# Integrated Transcriptome Profiling Revealed That Elevated Long Non-Coding RNA-*AC007278.2* Expression Repressed *CCR7* Transcription in Systemic Lupus Erythematosus

**DOI:** 10.3389/fimmu.2021.615859

**Published:** 2021-06-16

**Authors:** Yi You, Xingwang Zhao, Yaguang Wu, Jiangming Mao, Lan Ge, Junkai Guo, Chenglei Zhao, Dong Chen, Zhiqiang Song

**Affiliations:** ^1^ Department of Dermatology, Southwest Hospital, Third Military Medical University (Army Medical University), Chongqing, China; ^2^ Center for Genome Analysis, ABLife Inc., Wuhan, China; ^3^ Science Department, ABLife BioBigData Institute, Wuhan, China

**Keywords:** SLE, PBMCs, RNA-seq, lncRNAs, CCR7, Tfh cell differentiation

## Abstract

**Purpose:**

Systemic lupus erythematosus (SLE) is a serious autoimmune disease. Its molecular pathogenesis, especially the long non-coding RNA (lncRNA) function, remains unclear. We want to investigate the lncRNA dysregulation profile and their molecular mechanisms in SLE.

**Methods:**

In this study, we analyzed the transcriptome profiles (RNA-seq) of peripheral blood mononuclear cells (PBMCs) from SLE patients and two published transcriptome datasets to explore lncRNA profiles. The differentially expressed lncRNAs were confirmed by quantitative real-time PCR in another set of female patients. We constructed the lncRNA-mRNA regulatory networks by performing weighted gene co-expression network analysis (WGCNA). Dysregulated lncRNA AC007278.2 was repressed by short hairpin RNA (shRNA) in Jurkat cells. Dual-luciferase reporter gene assay was performed to investigate the regulatory mechanism of AC007278.2 on target gene CCR7.

**Results:**

We observed dominant up-regulation of transcripts, including mRNAs and lncRNAs, in SLE patients. By WGCNA method, we identified three modules that were highly related to SLE. We then focused on one lncRNA, *AC007278.2*, with a T-helper 1 lineage-specific expression pattern. We observed consistently higher *AC007278.2* expression in SLE patients. Co-expression network revealed that *AC007278.2* participated in the innate immune response and inflammatory bowel disease pathways. By knocking down *AC007278.2* expression, we found that *AC007278.2* could regulate the expression of inflammatory and cytokine stimulus response-related genes, including *CCR7*, *AZU1*, and *TNIP3*. *AC007278.2* inhibits the functional *CCR7* promoter to repress its transcription, thereby regulating autoimmunity and follicular T-helper cell differentiation.

**Conclusion:**

In summary, our study indicated the important regulatory role of lncRNAs in SLE. *AC007278.2* may be treated as a novel biomarker for SLE diagnosis and treatment.

## Introduction

Systemic lupus erythematosus (SLE) is a chronic autoimmune disease characterized by autoantibody production and multisystem inflammation. Studies in humans and experimental animal models have revealed a complex interaction of genetic and environmental factors in SLE, leading to immune dysregulation and immunological tolerance breakdown that, in turn, results in autoantibody production and multi-organ inflammation (1, 2). SLE pathogenesis is complex, and emerging evidence show that defective immune complex and biological waste clearance, neutrophil extracellular trap production, nucleic acid sensing, lymphocyte signaling, and interferon (IFN) production pathways contribute to a loss of tolerance and tissue damage (3). Recent insights into lupus pathogenesis has led to the development of new targeted therapies with more favorable side effect profiles (4). Although there have been many advances in the understanding of SLE pathogenesis, SLE etiology remains unclear. Investigation of the molecular mechanisms underlying dysregulation of immune response and autoimmunity initiation will facilitate the discovery of new therapeutic targets.

Genetic factors deeply influence the transcriptional and post-transcriptional changes in SLE patients and healthy people. Meanwhile, epigenetic modifications, including DNA methylation, histone modifications, and non-coding RNA regulation, play critical roles in regulation of gene expression in SLE patients (5, 6). Gene expression studies have contributed to the characterization of SLE pathogenic processes (6). Many studies have revealed the global transcriptional profile change in the peripheral blood mononuclear cells (PBMCs) of SLE patients using high-throughput sequencing methods (7–9). Multiple studies have revealed that dysregulation of type I IFN- and other cytokine-related genes are consistently observed in the PBMCs of active and severe SLE patients, indicating the decisive role of gene expression regulation in SLE pathogenesis (6, 8, 9).

Long non-coding RNAs (lncRNAs) are RNAs with no protein-coding feature, and are usually longer than 200 nucleotides (nt) (10). They play important roles in many aspects of the biological process, including embryonic development and differentiation (11), aging (12), and immune response (13, 14). Unlike small non-coding RNAs, lncRNAs have complex regulatory mechanisms, which are difficult to investigate owing to their multilateral known or novel functional mannerisms (15, 16). Recently, Zhao et al. summarized a new aspect of molecular regulation of lncRNAs in SLE (17). High-throughput methods have been used to explore lncRNA expression profiles and molecular functions in SLE (18–21). It was suggested that many lncRNAs influenced SLE pathogenesis by regulating target gene expression in cis- and/or trans-regulatory manners. However, further studies must be performed to investigate lncRNA functional mechanisms in SLE in detail.

To study the transcriptional changes in SLE patients systematically, especially with regard to lncRNAs, we performed whole transcriptome profiling of PBMCs by sequencing method (RNA-seq). Meanwhile, we also downloaded and integrated the published RNA-seq data from studies performed by Rai et al. (22) and Hung et al. (23) to perform a meta-analysis. The dominant up-regulation of both mRNAs and lncRNAs in SLE patients was observed in both sequenced and published datasets. Co-expression analysis revealed the potential lncRNA inflammatory functions. By exploring the functional effects of the highly up-regulated lncRNAs, including *AC007278.2*, in SLE, we found that *AC007278.2* regulated the inflammatory response-related gene expression in a trans-acting manner.

## Materials and Methods

### Extraction Method of PBMCs From the Blood Samples of SLE Patients

According to the SLE classification standards recommended by the American College of Rheumatology in 1997, patients with a high SLE Disease Activity Index (SLEDAI) were included, while those with infection, tumors, and other connective tissue diseases were excluded. This study was approved by the ethics committee of the First Affiliated Hospital of Army Military Medical University (KY2020167). Patients with SLEDAI were recruited according to SELENA-2K (24). Patients with SLEDAI <10 and 10 ≤SLEDAI ≤14 were included. Equivalent normal control cases with similar age range from the physical examination center of our hospital were included. Patients with abnormal blood routine, urine routine, and liver and kidney function and positive autoantibodies were excluded before inclusion. Detailed clinical and demographic characteristics of patients and health controls could be found in **Table S1**. The whole blood was collected in anticoagulant tubes (EDTA-K2, 2 ml, WEGO, China). The extraction procedure of PBMCs was performed as follows:

1. The whole blood collected in the anticoagulant tube was centrifuged at 100 rcf. Then, phosphate-buffered saline (PBS) solution (1 ml) was added to dilute the mixture to a ratio of 1:1 and mixed gently.
2. Ficoll (17144002, GE life) solution (5 ml) was added to the centrifuge tube (15 ml). The diluted blood (2 ml) was then added to the upper Ficoll layer in the centrifuge tube gently to ensure that the two solutions did not mix with each other and centrifuged at 400 rcf for 20 min. The deceleration setting was set to no breaking or only 1–2% breaking.
3. Draw off the upper layer, leaving the middle layer (PBMC) undisturbed at the interface. The middle layer of cells was sucked into another clean centrifuge tube (15 ml) using a clean Pasteur pipette. PBS (10–15 ml) was added, suspend the cells by gently drawing them in and out of a Pasteur pipette, centrifuged at 300 rcf for 10 min and remove the supernatant.
4. Resuspend cells in 2 ml of 1× Red Blood Cell Lysis Solution (BL503A, Biosharp), incubate 15 min at room temperature, centrifuged at 300 rcf for 10 min and remove the supernatant.
5. Three ml of PBS was added, suspend the cells by gently drawing them in and out of a Pasteur pipette, centrifuged at 100 rcf for 10 min and remove the supernatant. The cell pellet was lysed by trizol (15596-018, ambion) for RNA extraction.

### RNA Isolation, Library Construction, and Sequencing

Total RNA was treated with RQ1 DNase (Promega, USA) to remove DNA. The quality and quantity of the purified RNA were determined by measuring absorbance at 260 nm/280 nm (A260/A280) using SmartSpec Plus (BioRad, USA). RNA integrity was further verified by 1.5% agarose gel electrophoresis. For each sample, total RNA (10 µg) was used for RNA-seq library preparation. Polyadenylated RNA from two SLE patients and two healthy controls was purified and concentrated with oligo(dT)-conjugated magnetic beads (Invitrogen, USA) before directional RNA-seq library preparation. Purified mRNAs were iron-fragmented at 95°C, and end repair and 5’-adaptor ligation were performed. Then, reverse transcription (RT) was performed with the RT primer, which harbored the 3’-adaptor sequence and the randomized hexamer. The cDNAs were purified and amplified, and the polymerase chain reaction (PCR) products that corresponded to 200–500 base pairs were purified, quantified, and stored at −80°C until further use for sequencing.

For high-throughput sequencing, the libraries were prepared according to the manufacturer’s instructions and applied to the illumina X Ten System for 150 nt pair-end sequencing using Novogene (Beijing, China). The RNA-seq data were deposited in the NCBI Gene Expression Omnibus (GEO) database with accession number GSE139350.

### RNA-Seq Data Analysis

For RNA-seq data analysis, adaptors and low-quality bases were trimmed from the raw sequencing reads using Cutadapt (25) and reads shorter than 16 nt were discarded. Clean reads were aligned with the human-GRCH38 genome using TopHat2 (26), with no more than four mismatches. After mapping the reads onto the genome, we discarded the reads that were in multiple genomic locations due to their ambiguous origination. Reads with only one genome location were preserved to calculate the read number and reads per kilobase per million (RPKM) value for each gene. Differential expression between the SLE and normal control samples were analyzed using the edgeR package (27). For each gene, the p-value and the significance threshold to control false discovery rate (FDR) at a given value were calculated. Genes with FDR <0.05 and |log_2_ fold change| >1 were selected as differentially expressed genes (DEGs).

LncRNA prediction and analysis pipeline was followed the published study (28), which will not be described in this study. Differentially expressed lncRNAs were identified using the same method and criteria as DEGs.

We also downloaded the transcriptome data from two other studies (22, 23) that included the use of samples and methods similar to those in our study. GSE80183 dataset from Rai et al. (22) contained 16 samples, including 12 SLE samples and 4 controls from America. GSE72509 dataset from Hung et al. (23) contained 99 SLE samples and 18 controls from America, and we randomly selected 10 SLE samples and 5 controls to perform analysis. We re-analyzed the RNA-seq data by the same analysis pipeline and compared the results with those of this study to avoid individual variation.

### lncRNA-Knockdown Plasmid Construction

Short hairpin RNA (shRNA) clones of the gene of interest were designed by using the BLOCK-iT™RNAiDesigner online software (Thermo Fisher Scientific, United States). The silence sequence was 5’-TTGCCTTGATATGATTGTTGTT-3’. Sense and antisense strands were annealed to obtain the shRNA. Vector pGFP-B-RS was digested by HindIII and BamHI at 37°C for 2~3 h. Then, the enzyme-digested vector was run on 1.0% agarose gel and then purified using the Qiagen Column Kit. A linearized vector DNA was digested by HindIII and BamHI, and shRNAs were inserted using T4 DNA Ligase NEB. Plasmids were introduced into *Escherichia coli* bacteria by chemical transformation. Cells were placed on LB plates that contained kanamycin. The plates were then incubated at 37°C overnight. Colonies were screened by colony PCR (30 cycles) using universal primers (located on the backbone vector). The shRNA interference sequence was verified by Sanger sequencing.

### Cell Culture and Transfections

Jurkat cells (CL-0129) were obtained from Procell (Wuhan, Hubei, China). The cells were tested to be free of mycoplasma contamination. The cells were cultured in RPMI-1640 with 10% fetal bovine serum (FBS), streptomycin (100 µg/ml), and penicillin (100 U/ml) at 37°C in 5% CO_2_. Plasmid transfection of Jurkat cells was performed using Lipofectamine 2000 (Invitrogen, Carlsbad, CA, USA), according to the manufacturer’s instructions. Transfected cells were harvested after quantitative RT-PCR (RT-qPCR) analysis for 48 h. Glyceraldehyde-3-phosphate dehydrogenase (*GADPH*) was used as the control gene to assess the effects of *AC007278.2*-shRNA. cDNA was synthesized by using standard procedures, and RT-qPCR was performed on the Bio-Rad S1000 with Bestar SYBR Green RT-PCR Master Mix (DBI Bioscience, Shanghai, China) to assess *AC007278.2*-knockdown by shRNA. Information on the primers is presented in **Table S2**.

We obtained two biological RNA-seq replicate samples of *AC007278.2* for the knockdown experiment and analyzed these data with the same pipeline, as per previously described methods.

### RT-qPCR

To test the expression level change of the DEGs, differentially expressed mRNAs and lncRNAs (DElncRs) were randomly selected for RT-qPCR experiments to validate the DEG expression level. We selected PBMCs from five SLE patients and five healthy controls (**Table S1**), who did not participate in the RNA-seq experiments, to perform the RT-qPCR experiments. Polyadenylated RNA was enriched by using oligo(dT), as in the RNA-seq method, and then reverse transcribed into cDNA using M-MLV Reverse Transcriptase (Lot R011-01, Vazyme, Nanjing, China) and random primers. RT-qPCR was performed using the StepOneRealTime PCR System (QuantStudio™ 6 Flex Real-Time PCR System Contains the OptiFlex™ Optics System [Applied Biosystems™]) with the SYBR Green PCR Reagents Kit (Lot 11202ES08, Yeasen, Shanghai, China). The PCR conditions included denaturing at 95°C for 30 s, followed by 40 cycles of denaturing at 95°C for 10 s, and annealing and extension at 60°C for 30 s. PCR amplification was performed in triplicate for each sample and normalized to the human *GAPDH* gene. Data were assessed using the comparative threshold cycle (Ct) (ΔΔCt) method (29), and the relative expression level of the specific gene and *GAPDH* was thus obtained and presented. Three experimental replicates were performed for each sample. Primers for RT-qPCR analysis are listed in **Table S2**.

### Transfection and Luciferase Reporter Gene Assay

The 293T cells (10^5^ cells/ml) (Procell, China) were seeded in 96-well plates and grown in the Dulbecco’s Modified Eagle Medium supplemented with 10% FBS overnight. The predicted human *CCR7* promoter was amplified by PCR and subcloned into the pGL3-basic vector (Promega, USA). LncRNA *AC007278.2* was amplified by PCR and subcloned into the pCMV-Tag.2B-flag vector (Origene), and pRL-TK (Promega, USA) was co-transfected into the 293T cells using Lipofectamine 2000 (Invitrogen, 11668-027), according to the manufacturer’s instructions. After transfection for 48 h, the firefly and Renilla Luciferase activity was measured using the Dual-Luciferase Reporter Assay System (Promega, E1910).

### Co-expression and Weighted Gene Co-expression Network Analysis (WGCNA)

Based on the expression of each mRNA and lncRNA, Pearson’s correlation coefficient (PCC) and *p*-value were obtained for each mRNA-lncRNA pair. Then we filtered the result by a given threshold, with absolute correlation coefficient no less than 0.7 and P-value less than 0.01. The filtered mRNA-lncRNA pairs were used to construct expression network. To explore the gene expression pattern from all the DEGs, we used a weighted gene co-expression network analysis (WGCNA) method (30). The FPKM values of all identified DEGs, including mRNA and lncRNA genes, from the three datasets were combined together as the input file. We used default parameters of WGCNA to cluster these DEGs into different modules by their weighted correlation coefficients. For each module, eigengene was obtained to represent its expression pattern.

### Other Statistical Analyses

Principal component analysis (PCA) was used to analyze the lncRNA expression patterns. To predict gene function and calculate the functional category distribution frequency, Gene Ontology (GO) and enriched Kyoto Encyclopedia of Genes and Genomes (KEGG) pathway analyses were performed using the KOBAS 2.0 server (31). Hypergeometric test and Benjamini-Hochberg FDR controlling procedure were performed to define the enrichment of each pathway (corrected p-value <0.05). We compared the variance between the tested groups. Two-sided paired *t*-test was performed to calculate the DElncR number, and expression difference for individual genes. Hierarchical clustering was performed to cluster the mRNAs and lncRNAs with normalized values using the Cluster3.0. Java TreeView software (32). The star number represented the statistical significance: *p-value < 0.05; **p-value < 0.01; ***p-value < 0.001; ****p-value < 0.0001.

## Results

### RNA-Seq Results of the SLE and Normal Samples

**Figure 1:**
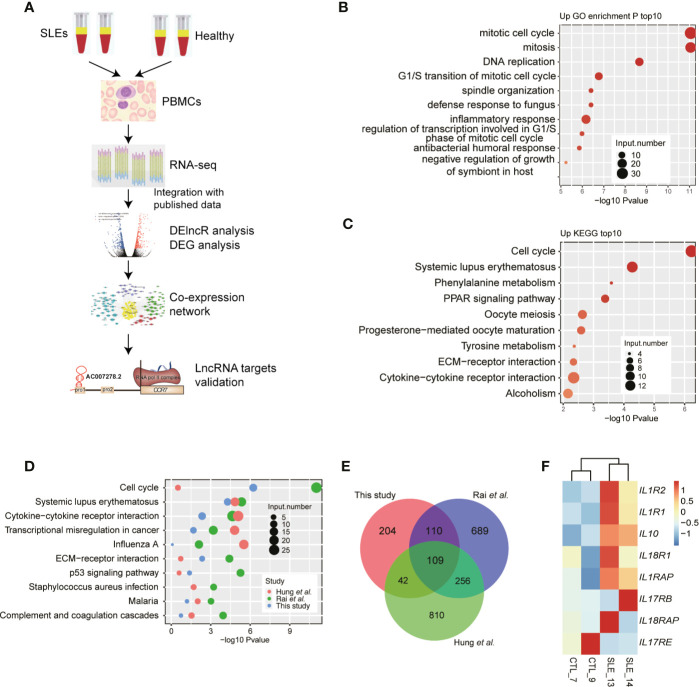
RNA-seq results show the aberrant protein-coding gene expression levels in systemic lupus erythematosus (SLE) peripheral blood mononuclear cell samples. **(A)** Workflow shows the experimental strategies and analyses in this study. **(B)** Bar plot shows the top 10 enriched Kyoto Encyclopedia of Genes and Genomes (KEGG) pathways of genes down-regulated in SLE patients. **(C)** Bar plot shows the top 10 enriched KEGG pathways of genes up-regulated in SLE patients. **(D)** Bubble plot shows the top enriched KEGG pathways of differentially expressed genes from three published datasets. **(E)** Venn diagram shows the overlap of up-regulated genes in the three RNA-seq datasets. **(F)** Heat map shows the up-regulation of interleukins and their receptors in SLE patients.

To explore the gene expression changes and their effects in SLE patients, we utilized a high-throughput sequencing method to perform transcriptome profiling (RNA-seq), to identify the aberrantly expressed genes, and then to explore and validate their functions by co-expression analysis (**Figure 1A**). As several studies using RNA-seq to explore gene expression difference in SLE patients have been published (8, 9, 22, 23) and could be reused to validate our discovery, it is not very necessary to re-performing RNA-seq experiments using large samples. Then we performed RNA-seq experiments using PBMCs from two SLE patients with high SLEDAI values (10 and 11) and two normal control individuals (**Table S3**). DEG analysis (|log_2_ fold change| ≥1 and FDR ≤0.05) showed more up-regulated genes (467 genes) than down-regulated genes (79 genes) in SLE patients (**Figure S1A**). We then performed DEG enrichment analysis using the functional annotations from the GO and KEGG databases. We found that cell cycle-related pathways were significantly enriched in the up-regulated genes (**Figures 1B, C**). The SLE pathway was also enriched in the up-regulated KEGG pathways (**Figure 1C**).

To compensate for the small sample size of this study, we also downloaded the published data from the studies by Rai et al. (22) and Hung et al. (23) to explore the DEGs from selected 16 and 15 RNA-seq samples, respectively. Consistent with our results, the results of the DEG analysis of the published data revealed that compared to down-regulated genes, more up-regulated genes were observed in SLE patients (**Figure S1B**). Functional enrichment analysis of the up-regulated DEGs from the three RNA-seq datasets showed similar enriched pathways, including the cell cycle and SLE pathways (**Figure 1D**) and the immune and inflammatory response-related terms (Figure S1C). Overlapping analysis of these three DEG sets also showed a significant overlap (**Figure 1E**), even though many DEGs were specifically detected in the three studies. The up-regulated genes included *IL10*, *IL1R1*, and *IL1R2*. By relaxing the threshold at p-value <0.05, we observed that seven interleukins (ILs) and their receptors were highly up-regulated in SLE patients, while only one IL receptor was down-regulated (**Figure 1F**). These eight ILs and their receptors also showed a higher expression in two published datasets used in this study (**Figures S1D, E**). Thereby indicating global transcriptome dysregulation in the SLE and healthy individuals.

### The Global lncRNA Expression Profile Was Highly Regulated in the SLE Patients

**Figure 2:**
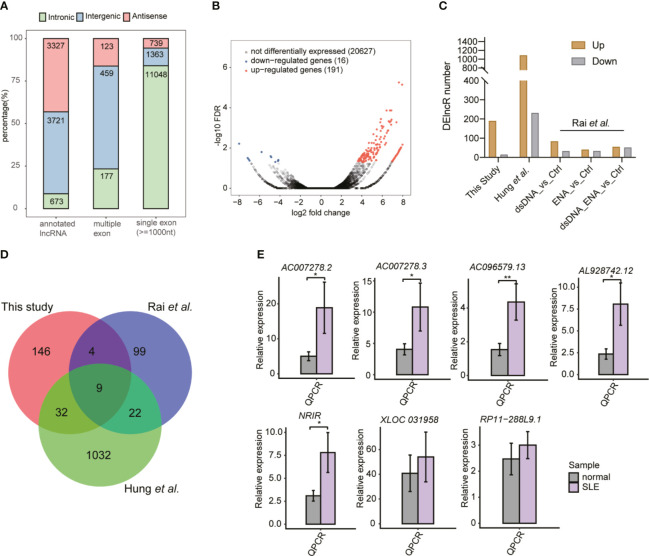
The global and altered long non-coding RNA (lncRNA) expression profile in systemic lupus erythematosus (SLE) patients and normal controls. **(A)** Bar plot shows the percentages of all kinds of lncRNAs in this study. **(B)** Volcano plot shows the dominant up-regulated lncRNAs in SLE patients, compared to the normal controls. **(C)** Bar plot shows the differentially expressed lncRNA (DElncR) number from the three datasets. RNA-seq samples from the study by Rai *et al.* were grouped into three pairs. **(D)** Venn diagram shows the overlapped DElncRs identified from these three RNA-seq datasets. **(E)** Bar plots show the RT-qPCR validation results of the seven selected DElncRs identified in this study (n = 5, mean ± standard deviation, mean ± SD). *p < 0.05; **p < 0.01; ***p < 0.001; two-tailed unpaired *t*-test.

The important roles of lncRNAs in SLE pathogenesis have been previously reported (17). To explore the global lncRNA expression pattern further, we used a cufflink-based transcript annotation pipeline (33) to predict lncRNAs and analyzed their expression profiles in SLE patients. We identified 13,082 novel lncRNAs after filtering the low-quality and potential protein-coding transcripts (see *Methods* for detailed information). Most of the predicted novel lncRNAs were located in introns and contained only one exon (**Figure 2A**). The predicted novel lncRNAs from the two published datasets showed similar classification features (**Figures S2A, B**). More predicted multiple- and single-exon lncRNAs were found to originate from intergenic and intronic regions, respectively (**Figures 2A** and **S2A, B**). PCA of all expressed lncRNAs exhibited separate profiles for the samples from these three datasets, thereby indicating the dominant effects of the batch on lncRNA expression (**Figure S2C**). Similar to the DEG results, the DElncR analysis results revealed that up-regulated lncRNAs in SLE were dominant (**Figure 2B**) (191 up-regulated and 16 down-regulated lncRNAs). The two published RNA-seq datasets also revealed more up-regulated lncRNAs in SLE patients (**Figure 2C**), thereby indicating the activated lncRNA expression pattern in SLE patients. Overlapping analysis of the DElncRs from these three datasets revealed that 67 lncRNAs in at least two datasets, including nine lncRNAs in three datasets, were up-regulated (**Figure 2D**), thus implying that the expression patterns of the DElncRs in the different datasets were more diverse than those of the DEGs. PCA analysis of the DElncRs also demonstrated separate profiles for the samples from these three datasets (**Figure S2D**). To validate the DElncR analysis results, we randomly selected seven up-regulated lncRNAs for RT-qPCR experiments. Although individual variation was observed, all of these seven lncRNAs (85.71%) showed consistently higher expression levels in SLE samples and five of them were statistically significant (**Figure 2E**). We also calculated their expression levels in the published dataset Rai et al. (22) and Hung et al. (23). Heatmap analysis showed that the elevated lncRNA expression levels in SLE patients were found to be consistent (**Figures S2E, F**). Taken together, these results indicated the regulated expression and potential regulatory roles of lncRNAs in SLE.

### Co-expression Analysis Revealed the Functions of DElncRs in SLE Patients

**Figure 3:**
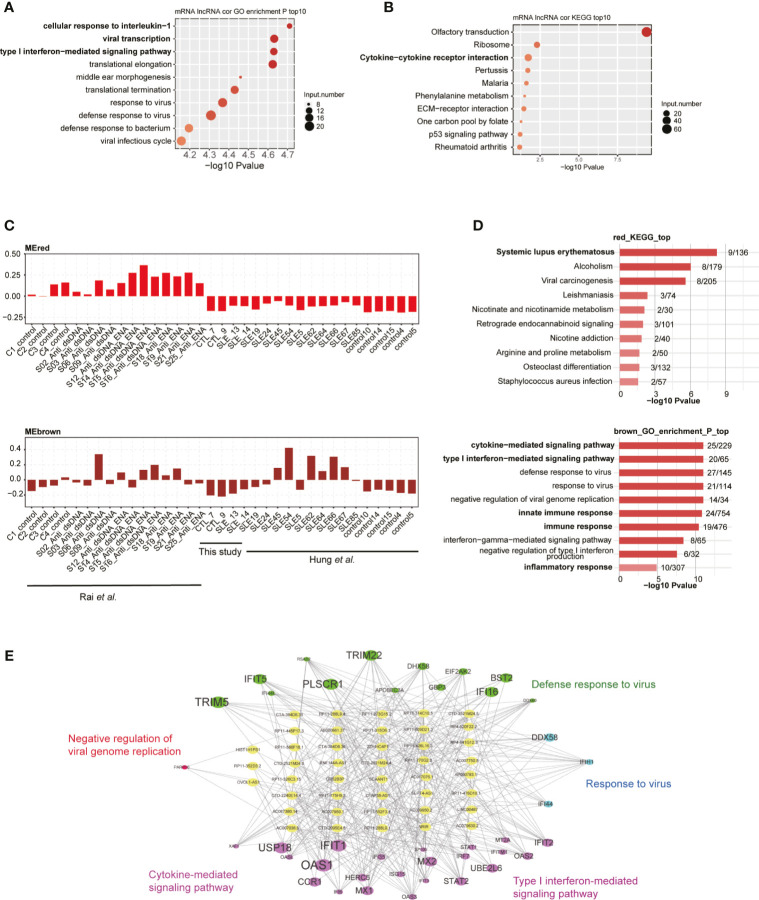
Long non-coding RNAs (lncRNAs) extensively interact with mRNAs in cis- and trans-acting manner. **(A)** Bubble plot shows the top 10 enriched biological process (BP) terms for genes that were found to interact with differentially expressed lncRNAs (DElncRs) by co-expression analysis. **(B)** Bubble plot shows the top 10 enriched Kyoto Encyclopedia of Genes and Genomes (KEGG) pathways for genes that were found to interact with DElncRs by co-expression analysis. **(C)** Bar plots show the eigengene values in red and brown modules. **(D)** Bubble plot shows the top 10 enriched BP terms for genes from the corresponding modules mentioned in **(C)**. **(E)** Regulatory network shows the potential interaction between lncRNAs and mRNAs from the brown module.

To explore the potential regulatory roles of lncRNAs, we performed the co-expression method to construct a network between lncRNAs and mRNAs. We used the two published datasets in our analysis pipeline to obtain more robust analysis results. By using absolute Pearson’s correlation coefficients (PCCs) >0.6 and *p*-value <0.05 as thresholds, we obtained more than 3,000 lncRNA-mRNA pairs, most of which (98.4%) were positively correlated (PCC >0). Functional analysis of these co-expressed mRNAs revealed that cellular response to IL-1, viral transcription, and type I IFN-mediated signaling were the three most enriched terms (**Figure 3A**), suggesting that these DElncRs played potential functional roles in SLE pathogenesis and/or development. We also detected that the viral/bacterial response-related genes were highly enriched in five of the top 10 terms (**Figure 3A**). KEGG analysis of these co-expressed mRNAs showed that olfactory transduction was the most enriched pathway. Cytokine-cytokine receptor interaction was the third most enriched pathway (**Figure 3B**). Most of these enriched pathways were highly related with SLE pathogenesis/development (34). Besides the co-expression profile, weighted gene co-expression network analysis (WGCNA) (30) was performed to explore the co-expressed lncRNAs and mRNAs. By analyzing all the DElncRs and DEGs from the three datasets, we obtained 20 co-expressed modules. The red, brown, and salmon modules showed higher expression levels of DEGs and DElncRs in the SLE samples from these three studies (**Figures 3C** and **S3A**). Functional analysis of the genes in these modules revealed that the genes were highly enriched in the systemic lupus erythematosus-, cytokine−mediated signaling pathway-, inflammatory response-, and innate immune response-related GO terms or KEGG pathways (**Figure 3D**). We selected lncRNA-mRNA pairs from the three modules for further analysis and found that the DElncRs were highly correlated with the DEGs that were found in the above-mentioned functional terms (**Figures 3E** and **S3B**). These co-expression analyses strongly suggested that DElncRs were the potential up-stream regulators of the activated immune and inflammatory response-related genes in SLE.

### One lncRNA Cluster Potentially Regulated Innate Immune Response-Related Gene Expression

**Figure 4:**
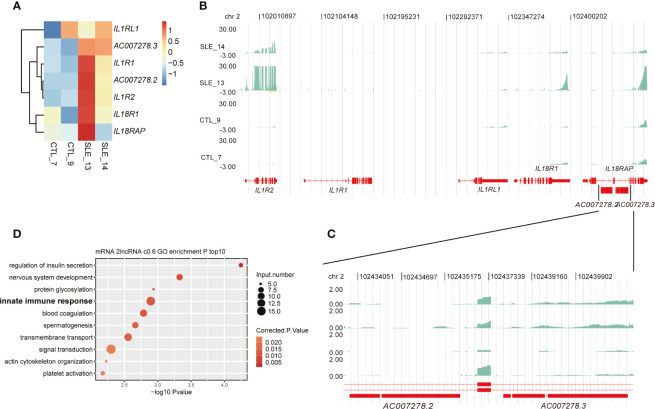
One lncRNA cluster potentially regulated innate immune response-related gene expression. **(A)** Heat map shows the elevated expression levels of seven genes in the systemic lupus erythematosus samples in this study. **(B)** Read density plot shows the global locus distribution and expression levels of these seven genes from the cytokine cluster. The two lncRNAs are amplified for a detailed view. **(C)** The detailed read density distribution of the two target lncRNAs *AC007278.2*/*AC007278.3* is shown. **(D)** Bubble plot shows the top 10 enriched Gene Ontology biological process terms of genes that were co-expressed with *AC007278.2*/*AC007278.3*.

Among these up-regulated lncRNAs in SLE, one lncRNA cluster, including two known multiple-exon lncRNAs *AC007278.2* and *AC007278.3*, was further investigated. Both RT-qPCR experiments and RNA-seq data showed elevated expression levels of these two lncRNAs in SLE patients (**Figures 2E**, **S2E, F**, **4A**), suggesting that these two lncRNAs were highly correlated (**Figure S4A**). Previous studies showed their lineage-specific expression patterns in primary and stimulated T-helper 1 (Th1) cells (35) and the association of their genomic variations with celiac disease (36). These two lncRNAs are located at Chr2 q11.2. This region contains five cytokine receptor genes, including *IL1R1*, *IL1R2*, *IL1RL1*, *IL18R1*, and *IL18RAP*. Expression analysis of these seven genes showed they were consistently elevated in SLE samples compared with control (**Figure 4A**). Two published datasets used in this study also revealed that these five cytokine receptor genes and two lncRNAs (*AC007278.2* and *AC007278.3*) showed higher expression levels in SLE patients (**Figures S4B, C**). We observed that the two lncRNAs are located within the intronic region of *IL18RAP* (**Figures 4B, C**). We then performed co-expression analysis to explore the potential targets of these two lncRNAs and detected 1,214 genes co-expressed *AC007278.2/AC007278.3* (PCC >0.6 and *p*-value <0.05). The co-expressed genes included *IL1R1* and *IL18RAP*. Functional enrichment analysis revealed that the co-expressed genes were enriched in the innate immune response term (**Figure 4D**). We suspected whether higher *AC007278.2* or *AC007278.3* expression triggered the expression of the adjacent cytokine receptor or other immune and inflammatory response-related genes in a trans-acting manner, thereby promoting SLE development.

#### *AC007278.2* Significantly Regulated the Inflammatory Response- and Cytokine Receptor-Related Gene Expression in a Trans-acting Manner

**Figure 5:**
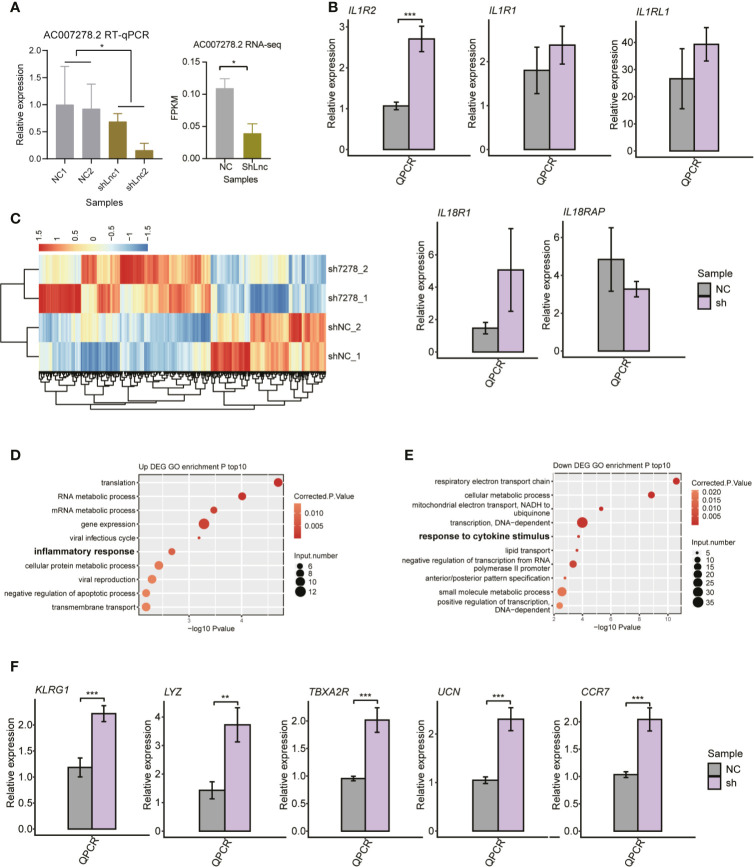
The transcriptional regulation of *AC007278.2* is observed by the knockdown experiment. **(A)** Bar plot shows the quantitative reverse-transcription polymerase chain reaction (RT-qPCR) experiment (left panel) and RNA-seq (right panel) results of repressed *AC007278.2* expression due to the short hairpin RNA (shRNA) knockdown experiment (mean ± SD). **(B)** Bar plot shows RT-qPCR experiment results of the five adjacent gene expression levels in the normal control and sh-*AC007278.2* samples (mean ± SD). **(C)** Hierarchical clustering heat map shows the differentially expressed genes regulated by *AC007278.2*. **(D)** Bubble plot shows the top 10 enriched Gene Ontology (GO) biological process (BP) terms for genes that were up-regulated by *AC007278.2* knockdown. **(E)** Bubble plot shows the top 10 enriched GO BP terms for genes that were down-regulated by *AC007278.2* knockdown. **(F)** Bar plot shows RT-qPCR experiment results of the five inflammatory response-related genes that were up-regulated by *AC007278.2* knockdown (mean ± SD). *p < 0.05; **p < 0.01; ***p < 0.001; two-tailed unpaired *t*-test. N.s. represented non-significant.

To characterize the *AC007278.2*/*AC007278.3* function of cytokine receptor regulation, we knocked down *AC007278.2* expression in Jurkat cells by the shRNA method. We chose only one of the lncRNAs because the two lncRNAs were highly correlated (**Figure S4A**). Compared with the control samples, significant decrease was observed in the *shAC007278.2* samples according to the RT-qPCR and RNA-seq results (**Figure 5A**). We then checked the expression level of the genomic-adjacent five genes, and found that it was not significantly regulated after *AC007278.2* knockdown except the most distant gene *IL1R2* (**Figure 5B**), thereby suggesting that *AC007278.2* perhaps regulated gene expression in a trans-acting manner. We then sequenced the transcriptome profiles (RNA-seq) of *shAC007278.2* and the control samples (**Table S3**), and identified the DEGs regulated by *AC007278.2*. Both the up- and down-regulated genes in the *shAC007278.2* samples showed consistent expression pattern in the two biological replicates (**Figure 5C**), according to the global regulatory pattern. We then analyzed the DEG functional terms enriched by *shAC007278.2*. Although they were differentially regulated by *AC007278.2*, the up- and down-regulated genes showed functional terms related to SLE pathogenesis (**Figures 5D, E**). Inflammatory and viral response-related genes were up-regulated, while cytokine stimulus response-related genes were down-regulated, suggesting that *AC007278.2* showed both positive and negative effects on immune response-related genes. The cytokine stimulus response-related down-regulated genes included *GNAO1*, *COL3A1*, *IL6ST*, *SKIL*, and *LIFR*. The inflammatory response-related up-regulated genes included *TBXA2R*, *LYZ*, *AZU1*, *TNIP3*, *CCR7*, *UCN*, and *KLRG1*. Inflammatory response is a biomarker and could lead to irreparable tissue damage of influenced organs in SLE progression (37, 38). We thus selected five inflammatory response-related genes to perform RT-qPCR experiments and found that all of them were significantly up-regulated in the *shAC007278.2* samples (**Figure 5F**).

#### *AC007278.2* Negatively Regulated *CCR7* Expression in SLE

**Figure 6:**
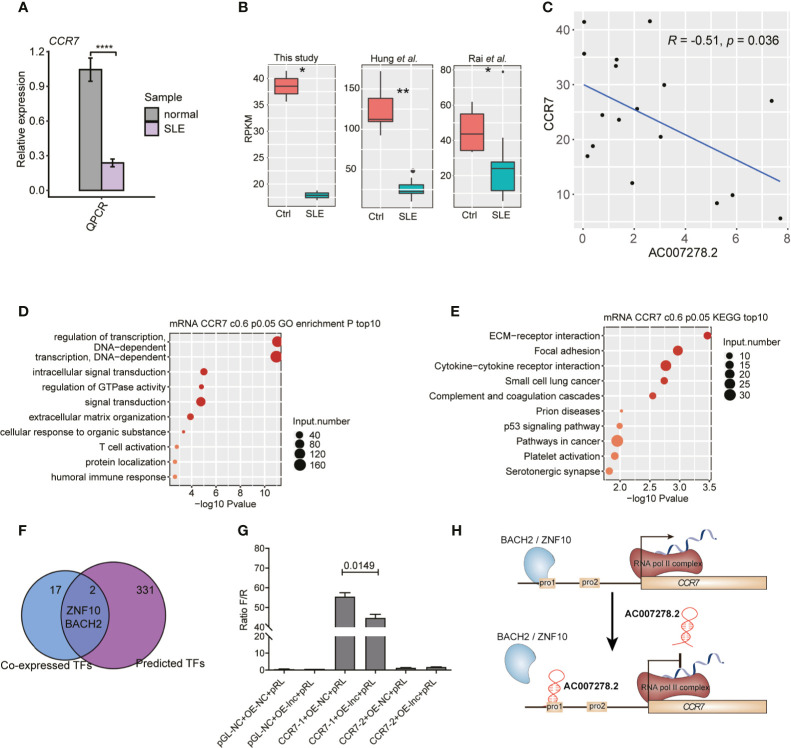
*AC007278.2* negatively regulates *CCR7* expression. **(A)** Bar plot shows quantitative reverse transcription-polymerase chain reaction (RT-qPCR) experiment results of increased *CCR7* expression level in SLE patients and normal controls (n = 5, mean ± SD). **(B)** Box plot shows the repressed *CCR7* expression level in systemic lupus erythematosus (SLE) patients from three datasets. **(C)** Dot plot shows the negative correlation between *AC007278.2* and *CCR7*. The line represents the regression line. **(D)** Bubble plot shows the top 10 enriched Gene Ontology biological process terms for genes that were co-expressed with *CCR7*. **(E)** Bubble plot shows the top 10 enriched Kyoto Encyclopedia of Genes and Genomes pathways for genes that were co-expressed with *CCR7*. **(F)** Venn diagram shows the overlapped co-expressed and GeneHancer-predicted transcription factors (TFs) for *CCR7*. **(G)** Bar plot shows the transcriptional repression function of *AC007278.2* on the first *CCR7* promoter (mean ± standard deviation, two-tailed unpaired *t*-test). PGL-NC represents empty plasmid; CCR7-1-OE and CCR7-2-OE represent CCR7 overexpression plasmid with first and second promoter, respectively (n = 3, mean ± SD). **(H)** The predicted working model based on the finding that *AC007278.2* negatively regulated *CCR7* expression is shown. *p < 0.05; **p < 0.01; ****p < 0.0001, two-tailed unpaired *t*-test.

Among the *AC007278.2*-regulated inflammatory response-related genes (**Figure 5D**), *CCR7* was found to be significantly up-regulated due to *AC007278.2* knockdown, and this expression change was validated by RT-qPCR (**Figure 5F**). We also validated the repressed expression of CCR7 in SLE patients by RT-qPCR (**Figure 6A**), and found that *CCR7* was significantly repressed in SLE patients from the two published datasets (**Figure 6B**), indicating an expression pattern inverse to that of *AC007278.2*. We then tested the correlated expression level of *AC007278.2* and *CCR7* and found that they were negatively correlated (**Figure 6C**). Previous studies have shown that *CCR7* plays an important role in primary immune response by establishing functional microenvironments in secondary lymphoid organs (39). *CCR7* ^−/−^ mice showed increased susceptibility to streptozotocin-induced diabetes and systemic autoimmune diseases, including SLE (40). The inverse expression pattern of *AC007278.2* and *CCR7* suggested that *AC007278.2* could induce SLE by repressing *CCR7* expression. We also analyzed the functions of the genes co-expressed with *CCR7*. Regulation of the transcription-related terms was in the top two GO biological process (BP) terms (**Figure 6D**). Extracellular matrix-receptor and cytokine-cytokine receptor interactions were among the top KEGG pathways (**Figure 6E**), and these results were consistent with those on *CCR7* functions. Transcription regulation-related genes might contain transcription factors (TFs) that regulate *CCR7* transcription. We then analyzed the TFs, co-expressed with *CCR7*, in the identified co-expressed genes (PCC >0.8). By integrating the enhancer prediction results from GeneHancer (41) and the co-expressed TFs, we detected two TFs, *ZNF10* and *BACH2*, which could regulate *CCR7* transcription (**Figure 6F**). These two TFs were down-regulated in SLE patients (**Figure S5A**), but their mRNA levels were not affected by *AC007278.2* knockdown (**Figure S5B**). To explore the *AC007278.2* regulatory mechanism of *CCR7*, we performed the dual-luciferase reporter gene assay. Two *CCR7* promoter sequences were extracted from the EPDnew databases. After transfection for 48 h, we found that the first *CCR7* promoter could considerably enhance transcription of the reporter gene, while the second *CCR7* promoter showed little effect (**Figure 6G**). After transfecting *AC007278.2*, the transcriptional level of the reporter gene, compared to that in the negative control, was significantly decreased (**Figure 6G**), thus revealing that *AC007278.2* could regulate *CCR7* transcription by affecting the function of the first *CCR7* promoter. Based on these results, we proposed a working model based on the finding that *AC007278.2* could restrict the binding of *ZNF10* or *BACH2* to the first *CCR7* promoter, thereby inhibiting *CCR7* transcription (**Figure 6H**).

## Discussion

In this study, we extensively analyzed the expression profiles of PBMC lncRNAs in SLE patients and healthy controls. The global up-regulated lncRNA profile in SLE patients showed alteration patterns similar to those of the mRNA profile. By analyzing one Th cell differentiation-associated lncRNA-*AC007278.2*, we found that this lncRNA could regulate the expression of inflammatory response-related genes, especially *CCR7*. These results extended the understanding of lncRNA functions in SLE pathogenesis and development.

The gene expression profile in SLE patients, compared to that in healthy individuals, was widely dysregulated (8). Recent studies also revealed the global lncRNA expression pattern and showed that thousands of lncRNAs were differentially expressed in SLE patients (19, 42), suggesting their potential regulatory roles (17). The dominantly elevated lncRNA expression pattern in SLE was observed in both this study and previously published studies. Based on their regulatory functions in the immune system, lncRNAs could contribute to the elevated expression of other protein coding genes in SLE (13). By integrating the published RNA-seq data, we found that lncRNAs were dominantly up-regulated in SLE patients, that showed changes similar to those in the protein-coding gene patterns. These results suggested that lncRNAs were globally dysregulated in SLE patients.

Unlike micro RNAs with well-known and canonical functional mannerisms (43), lncRNAs showed diverse functions that were difficult to predict (15, 44). The lncRNA functional mannerisms can be globally classified into two types: cis- and trans-acting (45). lncRNAs and their regulating targets may show positively or negatively correlated expression patterns (46). In this study, we performed co-expression analysis of lncRNAs and mRNAs to explore their potential interactive network. This method has been extensively used in studies on many biological and medical aspects, including development (47), aging (28), and disease (48). By integrating the published datasets, we identified over 3,000 lncRNA-mRNA interaction pairs, thereby indicating potential lncRNA regulatory functions in SLE. Over 95% of the co-expression pairs were positively correlated, thus highlighting the underlying transcription promotional roles of lncRNAs in SLE. By dividing this huge network into sub modules using the WGCNA method, we identified the modules that were related to immune and inflammatory response. Deregulated inflammatory factors play important roles in immune dysfunction and mediate organ damages in SLE patients (49). The activity score of SLE could be estimated by checking the expression level of pro-inflammatory factors in various organs, suggesting the clinical application values of inflammatory response genes (37). Several studies have demonstrated the regulatory factors in inflammatory response in SLE pathogenesis (50, 51). The lncRNAs co-expressed with inflammatory response-related genes in these modules were appropriate candidates to explore their biological functions in inflammation regulation for further studies.

Several lncRNAs in SLE, including *NEAT1*, *Gas5*, *MALAT1*, and *TUG1*, have been studied (17). A novel lncRNA, *lnc-MARCKS*, was recently reported to regulate inflammatory gene expression (52). In this study, we also focused on a function-unknown lncRNA, *AC007278.2*. A previous study only showed that *AC007278.2* was a Th1 lineage-specific lncRNA (35) with ambiguous functions. We observed consistently higher expression level of this lncRNA in SLE patients than in normal controls from multiple datasets, thereby indicating its potential regulatory functions in SLE. By knocking down its expression in Jurkat cells, the adjacent cytokine receptor genes were found to be not differentially expressed, thereby indicating the trans-functional manner of *AC007278.2*. We found that *AC007278.2* could significantly regulate inflammatory and cytokine stimulus response-related gene expression, thus confirming the biological functions of *AC007278.2*. *CCR7*, one of the targets of *AC007278.2*, has been proven to show significantly lower expression in SLE patients with active disease (53, 54). *CCR7*-deficient mice were prone to generalized multi-organ autoimmunity (40), which shared similarities with SLE-associated multi-organ involvement. These results suggested that *AC007278.2* promoted SLE development by repressing *CCR7* expression. However, *AC007278.2* did not regulate the expression of TFs that promoted *CCR7* expression; hence, we proposed that *AC007278.2* might repress *CCR7* transcription by blocking the onset of TFs in the *CCR7* promoter (**Figure 6H**). Several studies have reported the *CCR7* regulatory functions in follicular T-helper (Tfh) cell differentiation (55–57). Tfh cells promote SLE pathogenesis by promoting B-cell maturation and IL21 secretion (2, 58). Hence, we identified a connection between *AC007278.2* and SLE pathogenesis through the functional pathway that involved *AC007278.2*-induced *CCR7* expression down-regulation and Tfh cell promotion for B-cell maturation.

## Conclusions

In our study, we integrated the published RNA-seq datasets to illustrate the lncRNA expression profile in the PBMCs of SLE patients and normal controls. More importantly, we observed a functional link between *AC007278.2* and *CCR7* expression for SLE pathogenesis. We believe that our findings will help to understand the intact regulatory network of lncRNAs in immune cells and generate better experimental designs to investigate the lncRNA transcripts in SLE pathogenesis.

## Data Availability Statement

The datasets presented in this study can be found in online repositories. The names of the repository/repositories and accession number(s) can be found below: https://www.ncbi.nlm.nih.gov/geo/, https://www.ncbi.nlm.nih.gov/geo/query/acc.cgi?acc=GSE139350.

## Ethics Statement

The studies involving human participants were reviewed and approved by the Ethics Committee of Southwest Hospital. Written informed consent for participation was not required for this study in accordance with the national legislation and the institutional requirements.

## Author Contributions

YY, DC, and ZS conceived and designed the experiments. XZ, YW, JM, and LG participated in the sequence alignment and analyzed the data. JG and CZ performed the validation experiments. YY, XZ, and JM contributed to figure/reagents/materials/analysis tools. YY and ZS wrote the paper. All authors contributed to the article and approved the submitted version.

## Funding

This study was supported by National Natural Science Foundation of China (No. 81673058), Chongqing Basic Science and Frontier Technology Research (cstc2017jcyjAX0251). This study was also supported by ABLife Inc., Wuhan (No. 201801002) from DC.

## Conflict of Interest

Authors JM and DC were employed by the company ABLife Inc.

The remaining authors declare that the research was conducted in the absence of any commercial or financial relationships that could be construed as a potential conflict of interest.
