# Supplementary material for: Integrated Transcriptome Profiling Revealed That Elevated Long Non-Coding RNA-AC007278.2 Expression Repressed CCR7 Transcription in Systemic Lupus Erythematosus
Source: Front Immunol. 2021 Jun 16;12:615859. doi: 10.3389/fimmu.2021.615859 (PMC8242351; doi:10.3389/fimmu.2021.615859)
Supplement: Supplementary file 1 [file DataSheet_1.docx]

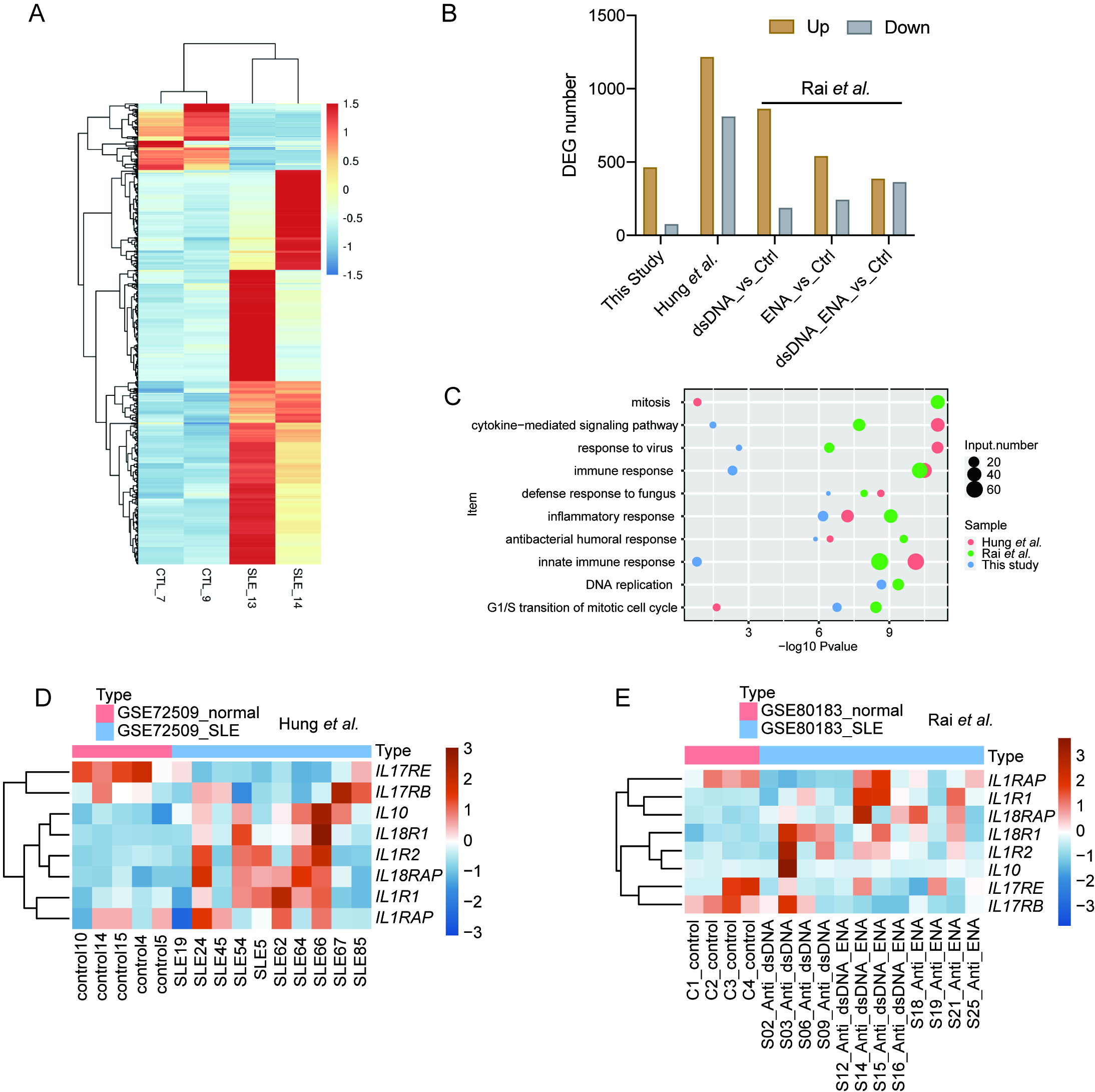


**Figure S1. The differentially expressed mRNAs and their functions in SLE patients. (A)** Hierarchical clustering heat map showed the expression pattern of DEGs between SLEs and health controls. (**B**) Bar plot showed the up and down DEG numbers in three studies. (**C**) Bubble plot showed the top enriched GO BP terms of up-regulated genes from these three studies. (**D**) Hierarchical clustering heat map showed the expression pattern of selected DEGs between SLEs and health controls from GSE72509 dataset. (**E**) Hierarchical clustering heat map showed the expression pattern of selected DEGs between SLEs and health controls from GSE80183 dataset.

**
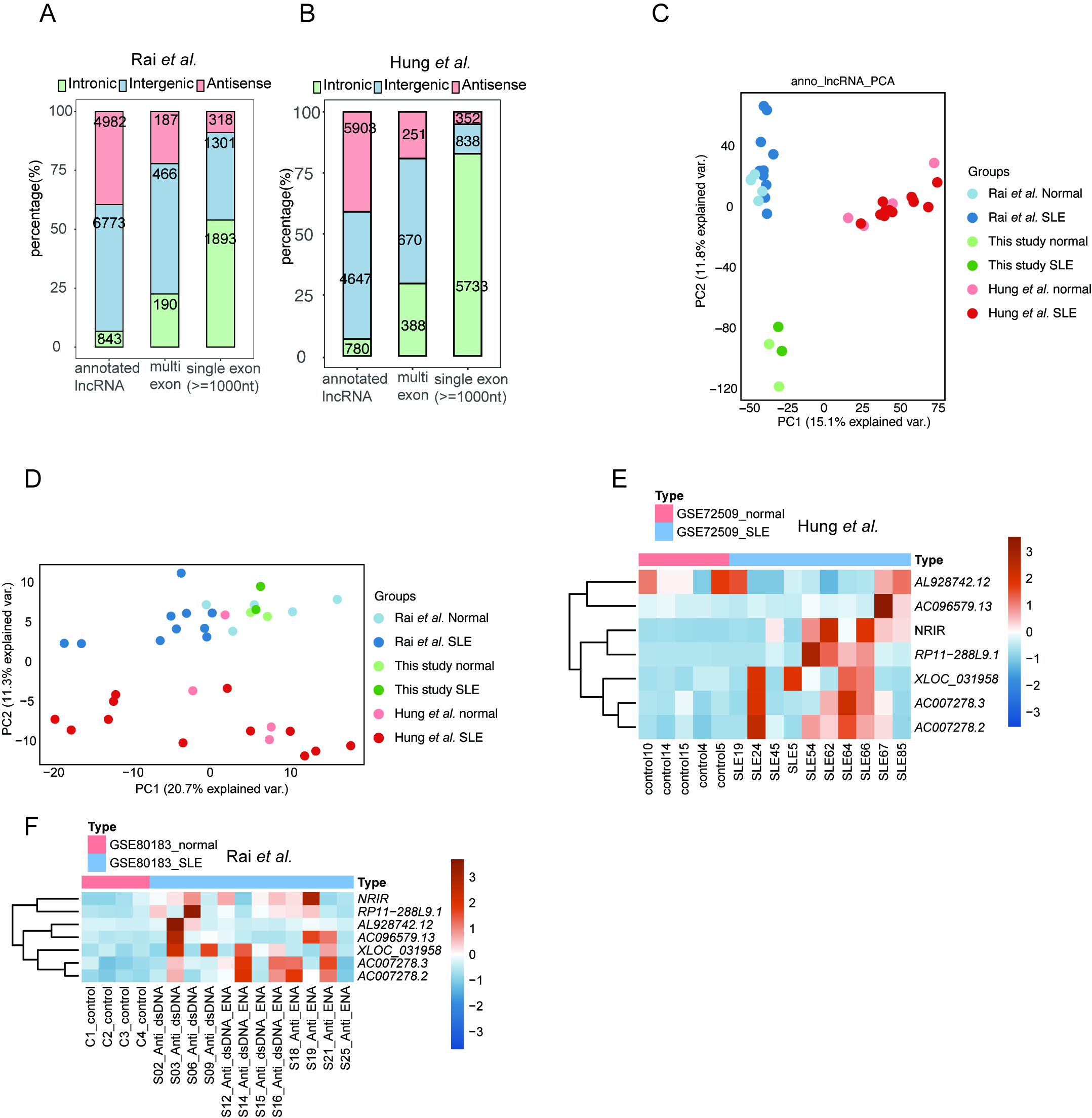
**

**Figure S2. The differentially expressed lncRNAs. (A-B)** Bar plot showing the percentage of all kind of lncRNAs in Rai *et al.* (A) and Hung *et al.* (B). (**C-D**) PCA results for all expressed lncRNAs (C) and the DElncRs (D). (**E**) Hierarchical clustering heat map showed the expression pattern of selected DElncRs between SLEs and health controls from GSE72509 dataset. (**F**) Hierarchical clustering heat map showed the expression pattern of selected DElncRs between SLEs and health controls from GSE80183 dataset.


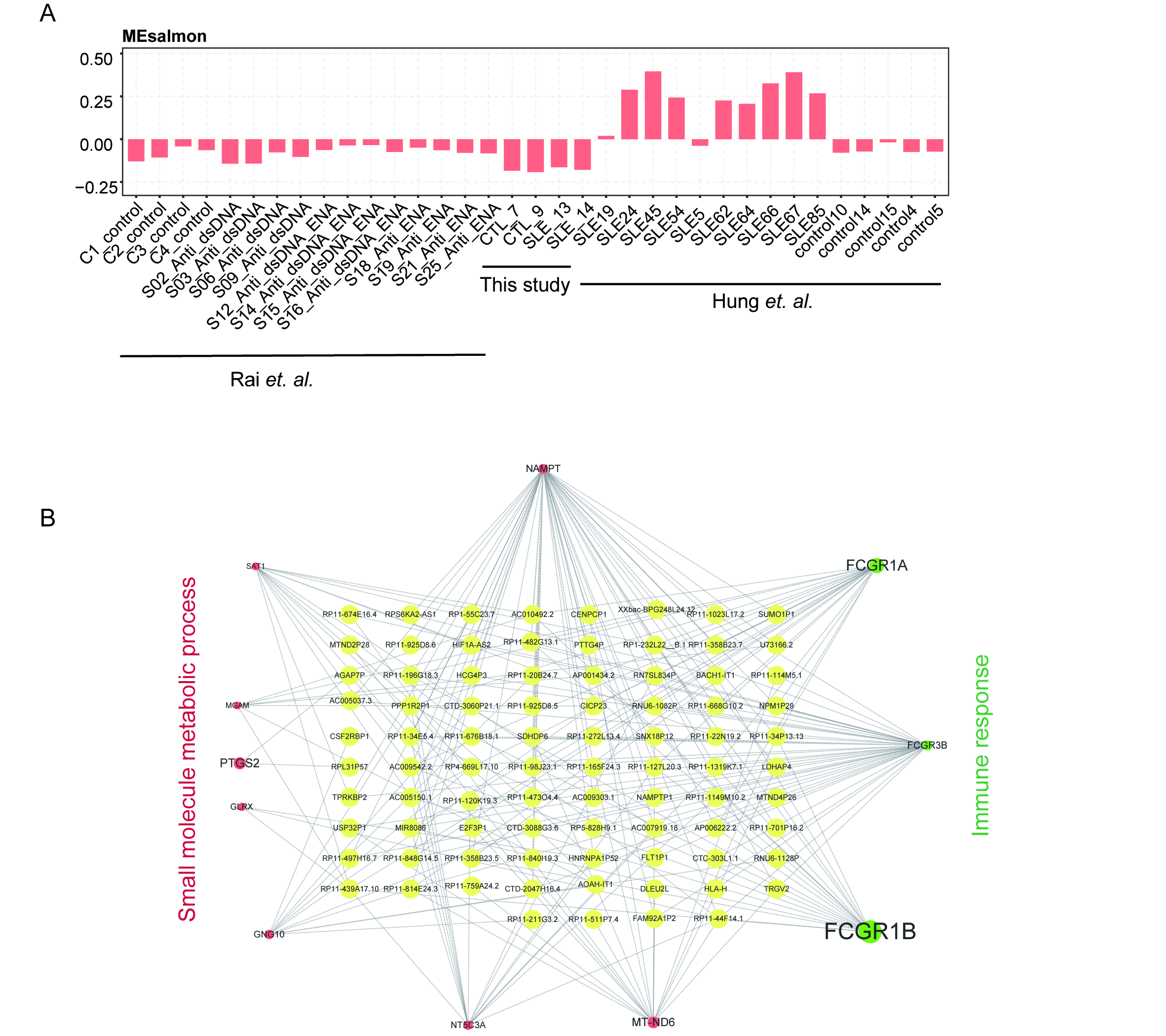


**Figure S3.** **LncRNAs were extensively interacted with mRNAs in cis-acting and trans-acting manner.** (**A**) Bar plots show the eigengene values in salmon modules. (**B**) Regulatory network showing the potential interaction between lncRNAs and mRNAs from red module.


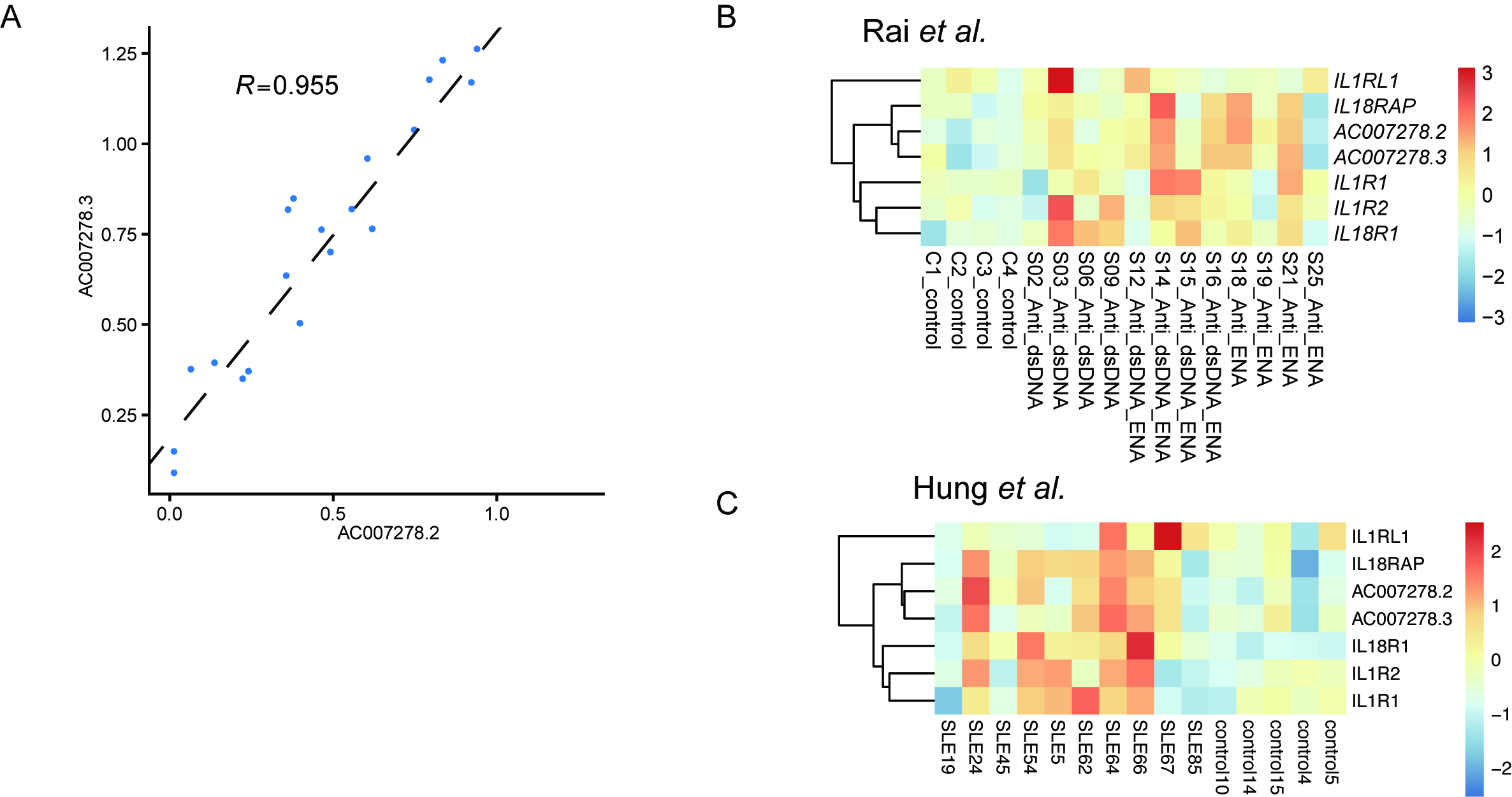


**Figure S4. The expression pattern of the five genes from the dataset of Rai et al and Bradley et al.** (**A**) Dot plot showing the highly correlation between *AC007278.2* and *AC007278.3*. (**B-C**) Heat map showing the elevated expression level of these seven genes in SLE patients in Rai *et. al.* (B) and Hung *et. al.* (C) datasets.


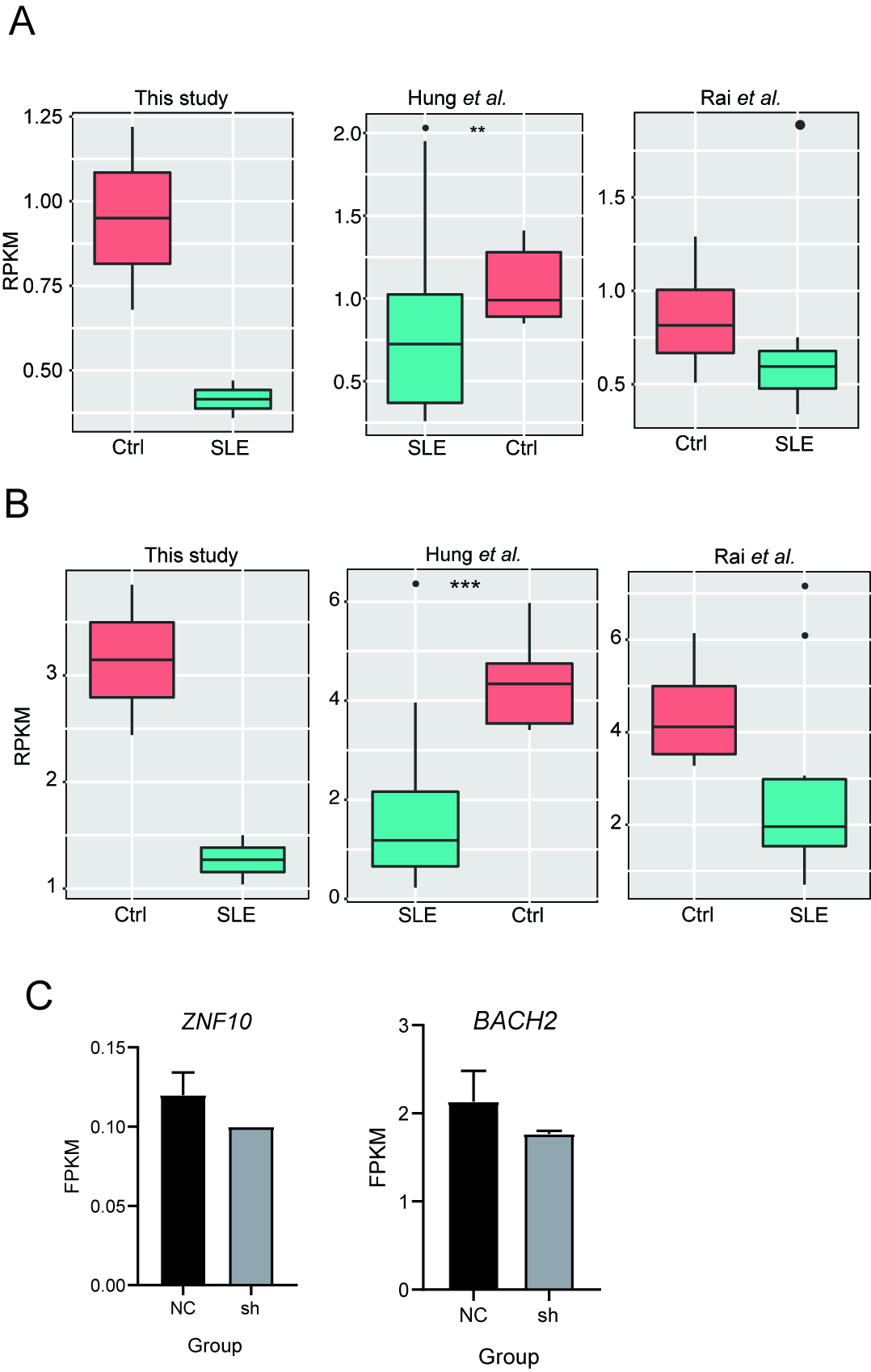


**Figure S5. The expression level of two related TFs in SLE patients and Jurkat cells.** (**A-B**) Box plot showing the repressed expression level of ZNF10 and BACH2 in SLE patients from the three datasets. (**C**) Bar plot showing the non-regulated expression level of ZNF10 and BACH2 in Jurkat cells by AC007278.2 knockdown experiment.

**Table S1. Clinical and demographic characteristics of the patients enrolled in this study.**

| **Sample** | **SLEDAI** | **Age** | **Sex** | **Purpose** |
| --- | --- | --- | --- | --- |
| **SLE 13** | 10 | 47 | Female | RNA-seq |
| **SLE 14** | 11 | 38 | Female | RNA-seq |
| **Control 7** | NA | 45 | Female | RNA-seq |
| **Control 9** | NA | 37 | Female | RNA-seq |
| **SLE-3** | 2 | 54 | Female | RT-qPCR |
| **SLE-16** | 9 | 43 | Female | RT-qPCR |
| **SLE-17** | 8 | 52 | Female | RT-qPCR |
| **SLE-18** | 10 | 15 | Female | RT-qPCR |
| **SLE-20** | 10 | 39 | Female | RT-qPCR |
| **Control-1** | NA | 38 | Female | RT-qPCR |
| **Control-2** | NA | 22 | Female | RT-qPCR |
| **Control-6** | NA | 25 | Female | RT-qPCR |
| **Control-13** | NA | 36 | Female | RT-qPCR |
| **Control-15** | NA | 33 | Female | RT-qPCR |

**Table S2. The primer sequences used for RT-qPCR experiment in this study.**

| **Genes** | **Forward** | **Reverse** |
| --- | --- | --- |
| *AC007278.2* | TCATCTGTATGCTGTCTAACCA | GATCTCATCTACATGCCTCACA |
| *AC007278.3* | TGTCCAATGCTGCTAAGAGGTA | GCCGCCATCTGTCCACTAA |
| *AL928742.12* | GCTGGAGGACGTTGGTGTA | CATCTTCGCCGCACTCTTC |
| *AC096579.13* | AGAGGAAGTTCACGGTCAGATA | ACTAAGCGATGACACCACACT |
| *XLOC_031958* | GAATCACCAGCAGTTGCCATAA | TTGCCTACTCTGACTCACCAC |
| *RP11-288L9.1* | GCGGAAGCCTGATGGTAACA | CCTCTGCCTGGAACACTTGT |
| *CCR7* | TCCTCTTCCTCCTGACCC | CTAGTATCCAGATGCCCACA |
| *IL1R1* | TGATGAATGTGGCTGAAA | TTGCTTGCCCAAGTATGT |
| *UCN* | CGGAGCTGTCCTGGCACTA | TGTCGGCGAGCGTCTGTA |
| *KLRG1* | GCTCCAACTACTCCACTT | TTTCCTGATTGTCCGTTA |
| *LYZ* | GAGATGTCCGTCAGTATGT | TTGCTCCTGCTTCTGTAA |
| *TBXA2R* | GACAGTGCTGCGAAACCCG | TGCTGAGGCGAGGCTGGAGA |
| *GAPDH* | GGTCGGAGTCAACGGATTTG | GGAAGATGGTGATGGGATTTC |

**Table S3. RNA-seq datasets used in this study.**

| **Study** | **Sample** | **Total Reads for Mapping** | **Total Mapped Reads (Percent)** | **Description** |
| --- | --- | --- | --- | --- |
| This Study | Cont 7 | 57558482 | 54211781(94.19%) | Health PBMC |
|  | Cont 9 | 54563714 | 50933066(93.35%) | Health PBMC |
|  | SLE 13 | 51359194 | 48014712(93.49%) | SLE PBMC |
|  | SLE 14 | 77345162 | 73177919(94.61%) | SLE PBMC |
|  | NC 1 | 99299150 | 96584406(97.27%) | Control Jurkat |
|  | NC 2 | 104064182 | 101060507(97.11%) | Control Jurkat |
|  | KD 1 | 98358706 | 95546220(97.14%) | Knockdown Jurkat |
|  | KD 2 | 71594800 | 69468993(97.03%) | Knockdown Jurkat |
| Rai *et al* | C1_control | 123410928 | 120287891(97.47%) | Health control |
|  | C2_control | 59393641 | 56108272(94.47%) | Health control |
|  | C3_control | 56290474 | 54292226(96.45%) | Health control |
|  | C4_control | 58182456 | 56461595(97.04%) | Health control |
|  | S02_Anti_dsDNA | 117645770 | 115468686(98.15%) | Anti-dsDNA+ SLE patient |
|  | S03_Anti_dsDNA | 71134944 | 68370318(96.11%) | Anti-dsDNA+ SLE patient |
|  | S06_Anti_dsDNA | 62941424 | 61326344(97.43%) | Anti-dsDNA+ SLE patient |
|  | S09_Anti_dsDNA | 82773328 | 80260561(96.96%) | Anti-dsDNA+ SLE patient |
|  | S12_Anti_dsDNA_ENA | 89233192 | 87142384(97.66%) | Anti-dsDNA+ENA+ SLE patient |
|  | S14_Anti_dsDNA_ENA | 75489218 | 73723533(97.66%) | Anti-dsDNA+ENA+ SLE patient |
|  | S15_Anti_dsDNA_ENA | 66376886 | 64576081(97.29%) | Anti-dsDNA+ENA+ SLE patient |
|  | S16_Anti_dsDNA_ENA | 93463304 | 91673732(98.09%) | Anti-dsDNA+ENA+ SLE patient |
|  | S18_Anti_ENA | 74646750 | 73085475(97.91%) | Anti-ENA+ SLE patient |
|  | S19_Anti_ENA | 66615482 | 64580879(96.95%) | Anti-ENA+ SLE patient |
|  | S21_Anti_ENA | 61459032 | 59249074(96.4%) | Anti-ENA+ SLE patient |
|  | S25_Anti_ENA | 65709058 | 64247803(97.78%) | Anti-ENA+ SLE patient |
| Hung et al | SLE19 | 92978547 | 92071227(99.02%) | SLE patient |
|  | SLE24 | 108120669 | 106894427(98.87%) | SLE patient |
|  | SLE45 | 110567949 | 109448947(98.99%) | SLE patient |
|  | SLE54 | 96579035 | 95311786(98.69%) | SLE patient |
|  | SLE5 | 86917464 | 86009243(98.96%) | SLE patient |
|  | SLE62 | 100088133 | 98928072(98.84%) | SLE patient |
|  | SLE64 | 122322742 | 121140318(99.03%) | SLE patient |
|  | SLE66 | 108886440 | 107757395(98.96%) | SLE patient |
|  | SLE67 | 93454358 | 92337415(98.8%) | SLE patient |
|  | SLE85 | 95064439 | 94057527(98.94%) | SLE patient |
|  | control10 | 94913405 | 94041483(99.08%) | Health PBMC |
|  | control14 | 101304880 | 100340489(99.05%) | Health PBMC |
|  | control15 | 104885284 | 103832001(99.0%) | Health PBMC |
|  | control4 | 106351881 | 105334564(99.04%) | Health PBMC |
|  | control5 | 91073383 | 90312540(99.16%) | Health PBMC |
